# Adhesion of voids to bimetal interfaces with non-uniform energies

**DOI:** 10.1038/srep15428

**Published:** 2015-10-21

**Authors:** Shijian Zheng, Shuai Shao, Jian Zhang, Yongqiang Wang, Michael J. Demkowicz, Irene J. Beyerlein, Nathan A. Mara

**Affiliations:** 1 Los Alamos National Laboratory, Los Alamos, NM 87545, USA; 2 Shenyang National Laboratory for Materials Science, Institute of Metal Research, Chinese Academy of Sciences; 3 School of Energy Research, Xiamen University, Xiamen 361005, China; 4 Department of Materials Science and Engineering, Massachusetts Institute of Technology, Cambridge, Massachusetts 02139, USA

## Abstract

Interface engineering has become an important strategy for designing radiation-resistant materials. Critical to its success is fundamental understanding of the interactions between interfaces and radiation-induced defects, such as voids. Using transmission electron microscopy, here we report an interesting phenomenon in their interaction, wherein voids adhere to only one side of the bimetal interfaces rather than overlapping them. We show that this asymmetrical void-interface interaction is a consequence of differing surface energies of the two metals and non-uniformity in their interface formation energy. Specifically, voids grow within the phase of lower surface energy and wet only the high-interface energy regions. Furthermore, because this outcome cannot be accounted for by wetting of interfaces with uniform internal energy, our report provides experimental evidence that bimetal interfaces contain non-uniform internal energy distributions. This work also indicates that to design irradiation-resistant materials, we can avoid void-interface overlap via tuning the configurations of interfaces.

Recently, high strength, outstanding thermal stability, and exceptional irradiation resistance have been achieved simultaneously by virtue of interface engineering in nanolayered materials^1^,^2^,^3^,^4^,^5^,^6^,^7^. It has also been established that interfaces can act as efficient sinks for point defects^8^,^9^. However, other radiation-induced defects, such as voids within the phases, can interact with the interfaces, reducing their cohesion. To effectively design bimetal interfaces to be simultaneously radiation resistant and mechanically strong, an understanding of interface interactions with voids at a fine scale is needed.

Generally, interfaces formed by joining two unlike solids are surfaces with characteristic energies, expressed in units of J/m^2^. The procedure for computing these energies, originally proposed by Gibbs^10^, yields a single average value for flat interfaces. However, many flat, solid-state interfaces have non-uniform internal structures. For example, semi-coherent interfaces consist of alternating regions of coherency separated by networks of inherent defects known as misfit dislocations^11^,^12^,^13^. Recent modeling investigations have shown that this structural non-uniformity leads to corresponding non-uniformity in interface energies, which are highest near misfit dislocation intersections (MDIs) and lowest within regions of coherency^14^,^15^,^16^. Precipitates forming at interfaces with location-dependent energies preferentially wet regions of high energy while regions with low energy might not be wetted at all^16^,^17^,^18^.

Because solid-state interfaces are buried within composite materials, their internal energy distributions are difficult to assess experimentally, especially when they vary over nanometer-scale distances. In this report, we show experimentally that interfaces between Cu and Ag lamellae contain non-uniform internal energy distributions. We find that, unlike with grain boundaries^19^,^20^,^21^, irradiation-induced voids do not overlap with bimetal interfaces. Instead, voids adhere to specific regions of these interfaces. They are also distributed asymmetrically, lying on the side of the phase with the lower surface energy, which is the Ag side at the Ag-Cu interfaces. This finding is consistent with predictions of non-uniform internal interface energy, which allows voids to wet some parts of the interface from one side of the interface, but not other parts. As we show here, atomistic molecular dynamics (MD) simulations support this interpretation.

## Results

### Adhesion of voids to Cu-Ag interfaces

**Figure 1:**
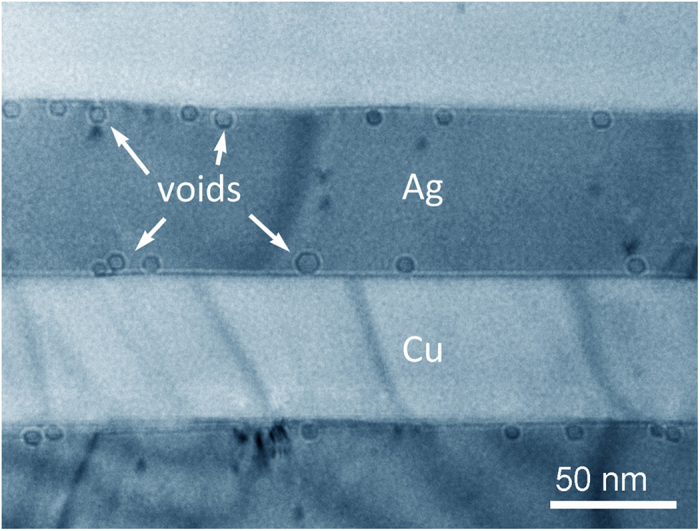
Adhesion of voids to Cu-Ag interface. Over-focus (+1.5 μm) TEM image of the Cu-Ag composite after He irradiation at 450 °C. Voids are represented by regions of dark contrast.

We begin with examination of the Cu-Ag nanolayered composite, where Ag has the lower surface energy than Cu. The Cu-Ag nanolayered composite with an eutectic composition (40–60 at.% Cu-Ag) was synthesized by a flux-melting technique^22^, and has about 30 nm and 65 nm thick Cu and Ag layers, respectively. Voids were induced via He irradiation on TEM foils of the Cu-Ag nanolayered composite (see Method and Supplementary information). Figure 1 shows a typical over-focus bright field transmission electron microscopy (TEM) image of voids within the Cu-Ag nanolayered composite after 200 KeV He-ion irradiation at 450 °C to a fluence of 2 ×10^17^. As shown, we find that the voids adhere to Cu-Ag interfaces from the Ag side. We also observe that the contact areas between the voids and the Cu-Ag interfaces are of nanometer-scale dimensions. This observation is unexpected since voids in single-phase polycrystalline metals normally overlap with the grain boundaries^19^,^20^,^21^.

### Surface wetting of interfaces with uniform energies

**Figure 2:**
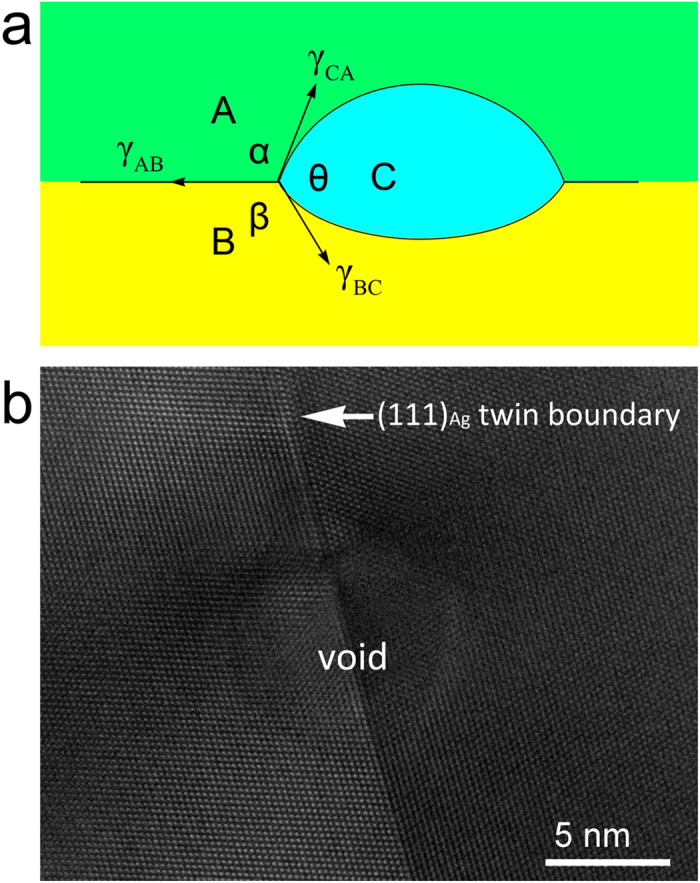
Surface wetting of interfaces with uniform energies. (**a**) Schematic of wetting on surfaces with uniform energy; (**b**) void wetting of a coherent (111)_Ag_ twin boundary.

To explain why the voids adhere to a small interface area on one side of the interface rather than overlapping the interface, we use arguments based on surface wetting^23^. As shown in Fig. 2a, when three adjacent phases (A, B and C) are in equilibrium, their surfaces meet at angles, α, β and θ, determined by ratios of their interface energies: γ_AB_, γ_AC_, and γ_BC_. The interface energies obey the geometrical constraint of 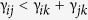, meaning no one interface energy can be larger than the sum of the other two. For this reason, when a precipitate forms at an interface, its equilibrium shape is usually lenticular and bulges slightly in the direction of the constituent with which it has a lower surface energy. This scenario assumes the interface energy γ_AB_ is uniform, i.e., it does not depend on location within the interface plane. For example, consider a void wetting a coherent (111)_Ag_ twin boundary. In this case, A and B are the same phase and the boundary has uniform energy. As shown in Fig. 2b, acquired from the same He-irradiated TEM sample as displayed in Fig. 1, a void overlaps symmetrically the twin boundary. However, semicoherent interfaces such as Cu-Ag and Cu-Nb are known to have non-uniform internal structure^24^,^25^,^26^. We therefore expect that they also have a non-uniform energy.

### Cu-Ag interface structures characterized by TEM

**Figure 3:**
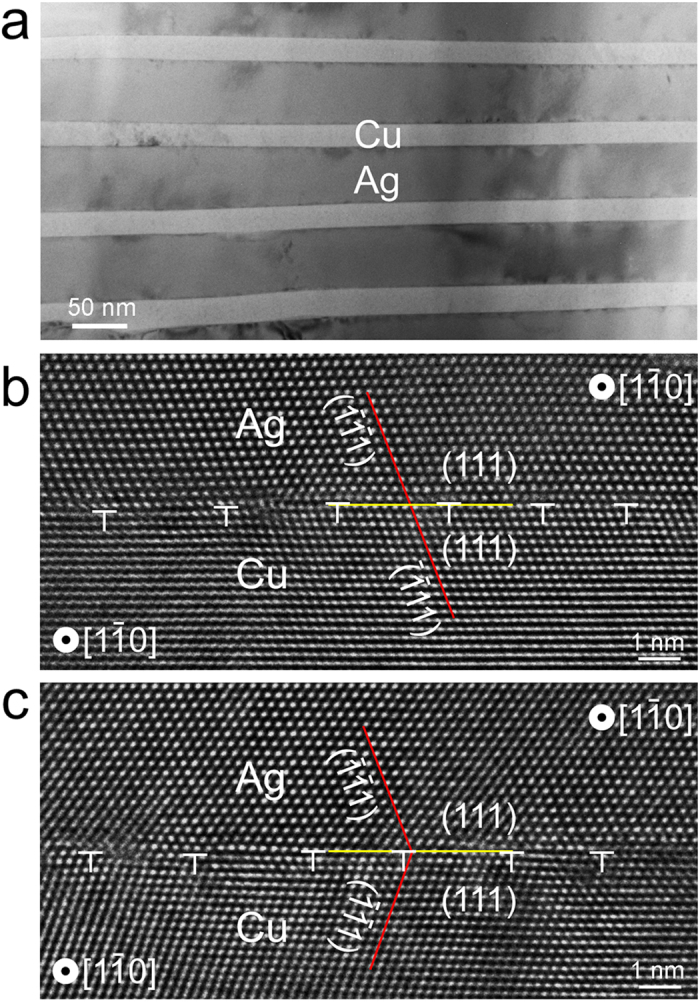
Cu-Ag interface structures. (**a**) TEM micrograph showing the Cu-Ag composite before He irradiation; (**b**) cube-on-cube and (**c**) hetero-twin Cu-Ag interface.

To investigate the location-dependent energy distribution in bimetal interfaces we will use atomistic modelling. However, building accurate atomic-scale models of interfaces requires first knowing the complete interfacial crystallography, i.e., misorientation and interface plane orientation^27^. For this reason, we perform high resolution TEM (HRTEM) analysis of individual interfaces. Figure 3a shows a typical TEM image of the as-prepared Cu-Ag composite. The Cu-Ag composite exhibits two types of interfaces, both possessing {111} interface planes. Type I interfaces have a cube-on-cube orientation relationship, where Cu and Ag have the same orientation across the interface, as illustrated in Fig. 3b. In type II interfaces, however, Cu and Ag exhibit a twin-like symmetry about the interface, as shown in Fig. 3c and hence we refer to type II interfaces as “hetero-twins”. This observation is consistent with previous studies on Cu-Ag interfaces via electron backscattered diffraction (EBSD)^28^ and TEM^29^. We also observe that the internal structures of the type I and type II interfaces are identical. This equivalence is consistent with our observation that both Cu-Ag interface types exhibit the same asymmetric void distribution.

### Direct observation of adhesion of voids to MDIs at Cu-Ag interfaces

**Figure 4:**
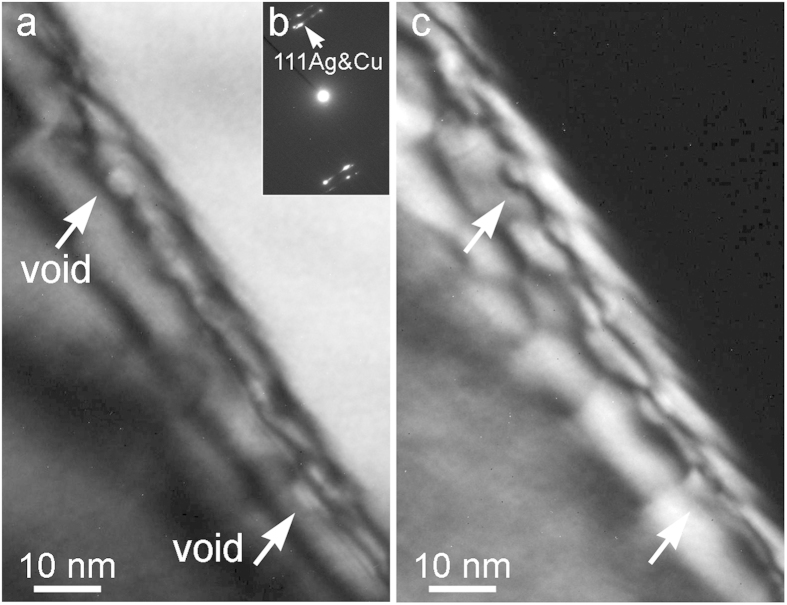
Adhesion of voids to MDIs at Cu-Ag interfaces. (**a**) Bright field TEM image and (**c**) dark field TEM image of a Cu-Ag interface under a two beam condition shown in (**b**) and at an under-focus of −1.5 μm.

Imaging under a two-beam condition has been used to identify voids at MDIs at Cu-Ag interfaces. Figure 4a,c are bright field TEM and dark field TEM images of the Cu-Ag interface under the two-beam condition shown in Fig. 4b. To perform the two-beam imaging, the sample was tilted so that the Cu-Ag interface overlapped and showed misfit dislocation patterns that are clear in the dark field TEM image in Fig. 4c. Voids located at MDIs are indicated by the arrows. These images provide direct evidence of adhesion of voids to MDIs.

### Non-uniform structures and energies of Cu-Ag interfaces studied by MD simulations

**Figure 5:**
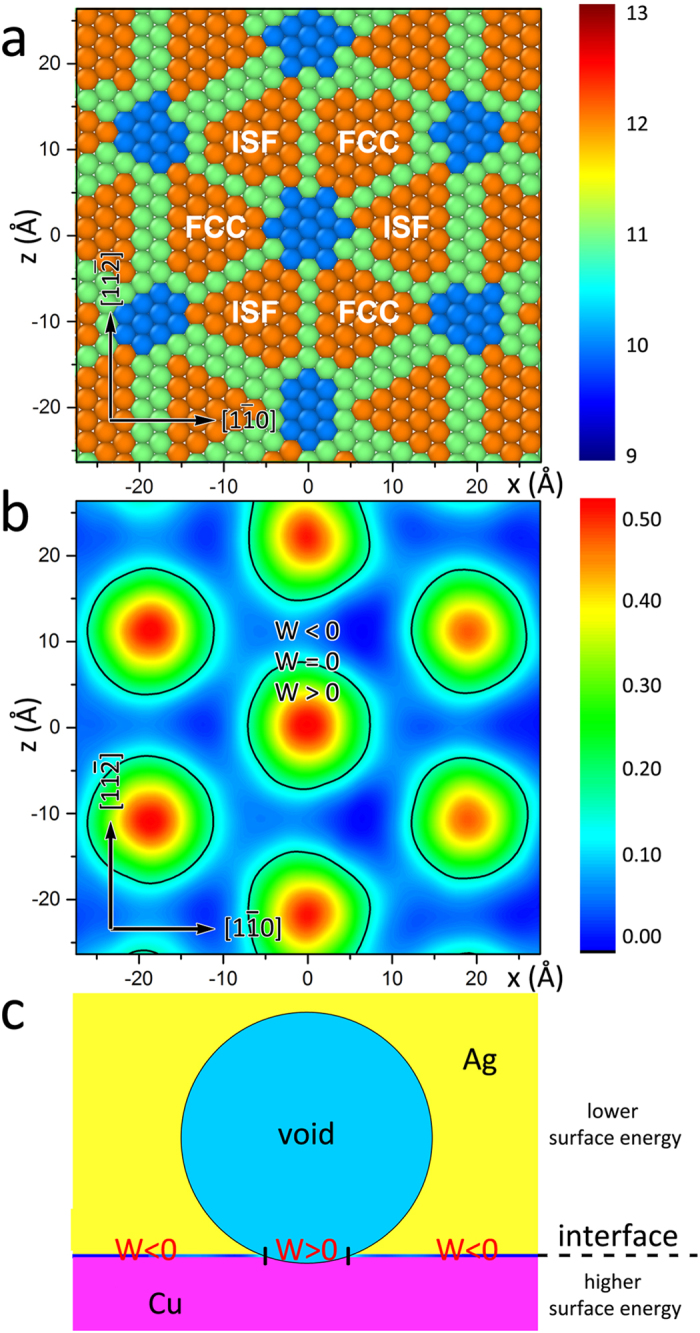
Void wetting of Cu-Ag interfaces with non-uniform structures and energies. (**a**) Misfit dislocation network in the cube-on-cube Cu-Ag interface. Atoms shown are on the Cu side of the interface and colored by coordination number. (**b**) Contour plot of the location-dependent interface energy of a Cu-Ag interface. Black contours correspond to zero wetting energy. (**c**) Schematic of a void wetting a single MDI where W > 0 at a Cu-Ag interface.

To explain our experimental findings, we characterize the internal structure of the Ag-Cu interface using MD simulations. By examining the coordination of interfacial atoms, we find that this interface contains three sets of misfit dislocations along the <110> directions, indicated by light-green atoms in Fig. 5a. The dislocation lines are Shockley partials with edge character^30^. All three sets of misfit dislocations intersect at periodic locations, shown by blue atoms in Fig. 5a. The misfit dislocation lines and intersections separate the coherent interface regions (orange atoms in Fig. 5a) containing perfect FCC stacking and intrinsic stacking faults (ISF). The coherent regions are associated with low potential energy and moderate coherency strain energy, while the dislocation lines and intersections have much higher energy density due to the cores of interface dislocations^24^. Therefore, a highly non-uniform interface energy landscape is expected. We computed the location-dependent interface energy on the Cu-Ag interface. As shown in Fig. 5b, the interface energy exhibits significant variations with location and these variations correlate to the misfit dislocation pattern of the interface. The coherent FCC stacking regions correspond to minima of the energy contour: they have energy of −0.02 J/m^2^. The energy of the coherent ISF regions is also low, having a slightly higher energy of 0.03 J/m^2^. The interface energy at dislocation lines is comparatively higher 0.06 J/m^2^. The MDIs correspond to maxima of the energy landscape and have a substantially higher energy of 0.47 J/m^2^. Such regular variations in interface energy have been predicted in other semi-coherent bimetal interfaces as well^25^,^31^.

### Void wetting of Cu-Ag interfaces with non-uniform energies

To explain void interactions with Cu-Ag interfaces, we use a wetting energy parameter, W = γ_A _+ γ_A-B_ − γ_B_, where γ_A_ and γ_B_ are the surface energies of phases A and B, and it is assumed that γ_A _< γ_B_. γ_A-B_ is the A-B interface energy^23^. When W > 0, thermodynamics favors wetting, meaning that the void will stay in A phase and touch the interface. In contrast, when W < 0, wetting is not favored and the void has minimum energy when it is entirely contained within the phase with the lowest free surface energy (the A phase). To calculate the surface energy associated with the formation of a void, we need to know the shape of the void. As discussed in the Supplementary information, voids in Cu and Ag assume the same truncated octahedron shape composed of {111} and {100} planes. We find that γ_Ag111 _= 0.92 J/m^2^, γ_Cu111 _= 1.06 J/m^2^, γ_Ag100 _= 0.99 J/m^2^, and γ_Cu100 _= 1.13 J/m^2^ 32. Consequently, with all else being the same, the void will have a smaller surface energy in Ag than Cu. If the void wets the {111}_Cu_-{111}_Ag_ interface from the Ag (A phase) side, the wetting results in a {111}_Cu_ surface replacing an equal area of {111}_Cu_-{111}_Ag_ interface and {111}_Ag_ surface. In this case, the wetting parameter is calculated as: W = γ_Ag111 _+ γ_Cu-Ag_ − γ_Cu111_. Because γ_Cu-Ag_ varies from location to location within the interface, therefore so does W and the likelihood for void touching.

Figure 5b shows regions with W > 0, which occur at MDIs, and W < 0, located at coherent patches. The black contour denotes W = 0. Voids completely wet regions where W > 0. Beyond the black contours, however, where W < 0, the voids do not wet the interface at all. Within the wetting area, W > 0 is consistent with the geometrical constraint 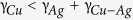, given by Neumann’s triangle^33^. Since the regions where W > 0 are small, the curvature deviation from {111}_Cu_ surface is too small to be observed by TEM. However, once a void has grown large enough to cover an entire MDI, it is not thermodynamically favorable for it to continue to wet the interface as it grows. Instead, it extends into the side with lower surface energy, i.e., the Ag phase, as illustrated in Fig. 5c. Then it can become visible in the TEM. This growth process results in a different equilibrium void shape than that expected on an interface with uniform energies. The notion of non-uniform interface energies explains the asymmetric void distribution about bimetal interfaces reported here.

## Discussion

Previous simulations have found that similar wetting arguments to those given above may also be used to explain the formation of He precipitates at Cu-Nb interfaces with a Kurdjumov-Sachs orientation relationship (KS Cu-Nb)^16^. Our investigation is consistent with this previous simulation work. However, no direct experimental validation of this prediction was previously available. The present work provides such validation through the TEM observations of Cu-Ag interfaces shown in Fig. 1. Although the Cu-Ag and KS Cu-Nb interfaces have different interface structures and orientations^29^, they show the same void distribution phenomenon indicating the wetting controlled void distribution is universal at bimetal interfaces. The conditions governing interface void formation are important since the configuration of voids and bubbles significantly influence the properties of irradiated materials^17^,^34^. Moreover, by controlling the internal structure of bimetal interfaces, it may be possible to tailor the way they interact with voids and other precipitates^35^. For example, interfaces may be used as precipitation templates for implanted impurities, reducing the damage caused by such impurities^36^.

In summary, using TEM we have demonstrated that voids distribute at hetero-interfaces asymmetrically. The asymmetric void distribution can be rationalized based on the phase with the lower surface energy and wetting of interfaces with heterogeneous formation energies. These findings can provide insight into designing irradiation-resistant materials. Voids that just touch the interfaces may be less harmful to cohesion than those that overlap the interfaces because the former will give rise to a smaller reduction of interface bonded area than the latter. Optimization could include choosing proper constituents of the composites and tuning their interface energies by adjusting their crystallography such that void-interface overlap or even touching is hindered.

## Methods

### Materials fabrication

The bulk nanolayered Cu-Ag composite with an eutectic composition (40–60 at.% Cu-Ag) was fabricated via a flux-melting technique^22^. The starting materials used for the eutectic preparation are Ag (99.999% pure) and Cu (99.999% pure) fragments. Mixtures of the starting materials were placed in fused silica tubes together with pieces of B_2_O_3_ flux. The tubes were then heated slowly to above 1200 °C to melt the B_2_O_3_, Ag, and Cu. When the flux melting was completed, the fused silica tubes containing the melt and the B_2_O_3_ flux were quenched into water. The diameter and length of the ingots are about 8 mm 50 mm, respectively.

### TEM characterization

TEM samples were prepared by a conventional cross-sectioning method, consisting of low-speed saw cutting, mechanical polishing, dimpling, and ion milling on a Gatan precision ion polishing system (PIPS) operated at 3.5 kV. TEM was performed on a Cs-corrected Titan 80–300 (FEI) operated at 300 kV.

### He ion irradiation

He ion irradiation was conducted using a Danfysik 200 kV ion implanter at Los Alamos National Laboratory. The TEM samples were mounted with silver paste onto a large Cu holder, through which the irradiation temperatures were controlled. Additionally, the He ion beam was perpendicular to the TEM samples. To generate voids in the TEM observation available regions of TEM samples (generally with thickness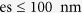), high enough energy (200 keV) and fluence (2 × 10^17^ ions/cm^2^) according to SRIM calculation^37^, as well as high temperature 450 °C were selected^2^. In this setting, a damage level of ~3 displacements per atom (dpa) was produced in Cu, Ag, and Nb. Moreover, to confirm that these cavities are voids, the distribution of He concentration in the TEM samples have been calculated by SRIM^37^, and low-temperature irradiation experiments with the same He ion energy and fluence were performed (Supplementary information).

### Atomistic modelling and analyses of the Cu-Ag interface

We use molecular dynamics simulations to obtain the Cu-Ag (111) interface structure. The Cu-Ag (111) bilayer model with cube-on-cube orientation relation is first constructed by joining two rectangular single crystals of Cu and Ag together. The orientations for both crystals are the same: x-axis along 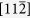, y-axis along [111] and z-axis along 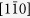. The interface is perpendicular to the y direction. Periodic boundary condition (PBC) are applied in the x and z directions, and a semi-fixed boundary condition is applied in the y direction^14^. To minimize the internal stress created by the imposition of PBCs, model dimensions are chosen to be 19 nm, 10 nm and 11 nm in the x, y and z directions. We modelled interatomic interactions using embedded-atom method (EAM) potentials for Cu, Ag, and their cross pair^30^,^38^. Such potentials have been shown to produce reliable interface properties^14^,^24^,^39^. The structure is then relaxed using quenching molecular dynamics^40^. Each layer of the equilibrium structure has zero stress in the y direction, and less than 10 MPa in the x and z directions.

We analysed the relaxed Cu-Ag interface model by performing coordination number and energy calculations at the interface. The coordination number of an interfacial atom is the number of atoms within a cut-off radius (rcut). The cut-off radius is chosen as the average of the first and second nearest neighbour distances in bulk conditions. For instance, the cut-off radii used for Cu and Ag atoms are 0.309 nm and 0.349 nm, respectively. We computed the location-dependent interface energy on an array consisting of 91 and 51 points along the x and z directions of the interface, respectively. For each sampling point, local interface energy is calculated within a cylinder straddling on the interface with radius r = 0.5 nm and height h = 7 nm. The axis of the cylinder is parallel to the interface normal; the geometric center of the cylinder coincides with the sampling point on the interface. The local interface energy is calculated according to 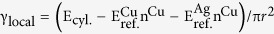, where n_Cu_ and n_Ag_ are the number of Cu and Ag atoms in the cylinder. 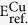 and 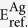 are the cohesive energies per atom of Cu and Ag.

## Additional Information

**How to cite this article**: Zheng, S. *et al.* Adhesion of voids to bimetal interfaces with non-uniform energies. *Sci. Rep.* **5**, 15428; doi: 10.1038/srep15428 (2015).

## Supplementary Material

Supplementary Information
