# Supplementary material for: Adhesion of voids to bimetal interfaces with non-uniform energies
Source: Sci Rep. 2015 Oct 21;5:15428. doi: 10.1038/srep15428 (PMC4614025; doi:10.1038/srep15428)
Supplement: Supplementary Information [file srep15428-s1.pdf]

Adhesion of voids to bimetal interfaces with non-uniform energies

Supplemental Information

S.J. Zheng[1,2], S. Shao[1], J. Zhang[1,3], Y.Q. Wang[1], M.J. Demkowicz[4], I.J. Beyerlein[1], and N.A. Mara[1]

[1] Los Alamos National Laboratory, Los Alamos, NM 87545, USA.

[2] Shenyang National Laboratory for Materials Science, Institute of Metal Research, Chinese Academy of Sciences.

[3] School of energy research, Xiamen University, Xiamen 361005, China.

[4] Department of Materials Science and Engineering, Massachusetts Institute of Technology, Cambridge, Massachusetts 02139, USA.

Correspondence and requests for materials should be addressed to S. Z. (sjzheng@imr.ac.cn).

1. Voids but not He bubbles produced by irradiation

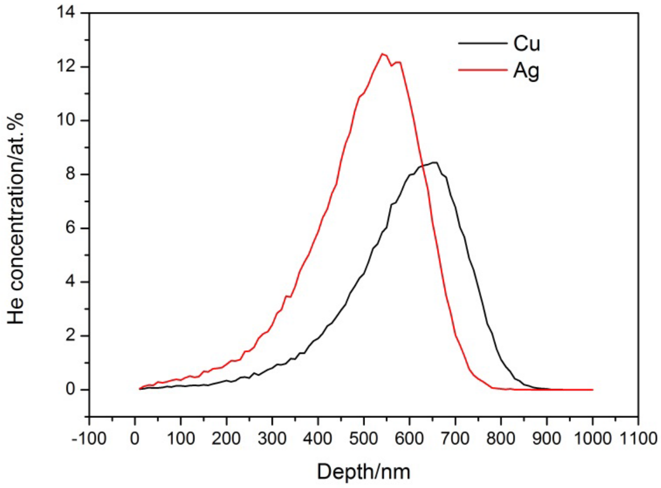

Supplementary Figure 1: He concentration in Cu, Nb, and Ag simulated by SRIM with setting of “Detailed Calculation with full Damage Cascades”. Generally, thickness of observable region in a TEM sample is less than 100 nm, thus the average He concentrations in such regions of Cu and Ag are 0.09 at.% and 0.24 at.%, respectively. Additionally, the calculations are performed in layers with 1000 nm thickness which are ten times thick of the TEM samples and contain bulk effects, also temperature effect is not considered, therefore the real He concentrations in the TEM samples should be much smaller than the values given above.

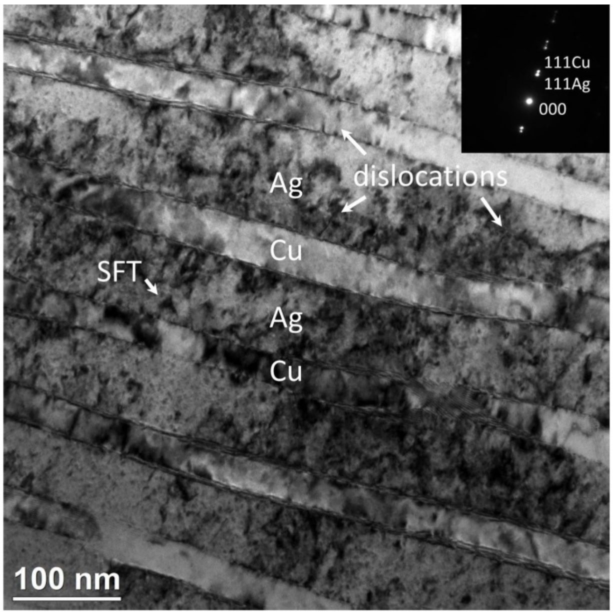

Supplementary Figure 2: defects such as dislocations and stacking fault tetrahedrons, not voids, caused by He irradiation at room temperature.

A TEM image of the Cu-Ag composite after He irradiation at room temperature. As demonstrated by the inset, which is the corresponding electron diffraction pattern, the TEM image was taken at an approximate two beam condition. Numerous irradiation-induced defects

such as dislocation loops and stacking fault tetrahedrons (SFTs) may be seen. However, no voids are formed.

Considering the small He concentration and the defects generated during He irradiation at room temperature, it can be induced that the cavities found after He irradiation at 450 ºC are voids. If He concentrate is high enough, it would have precipitated into nanoscale bubbles at room temperature due to its insolubility in Cu, Nb, and Ag. Additionally, according to our previous simulations [16], cavities found after He irradiation at 450 ºC which are about 6 nm in diameter are big enough to form stable voids without He. Moreover, even if there is any He in such cavities, its pressure is low enough to be ignored. Thus, we think it more accurate to refer to the cavities as voids.

2. Truncated octahedron shape of ion-induced voids

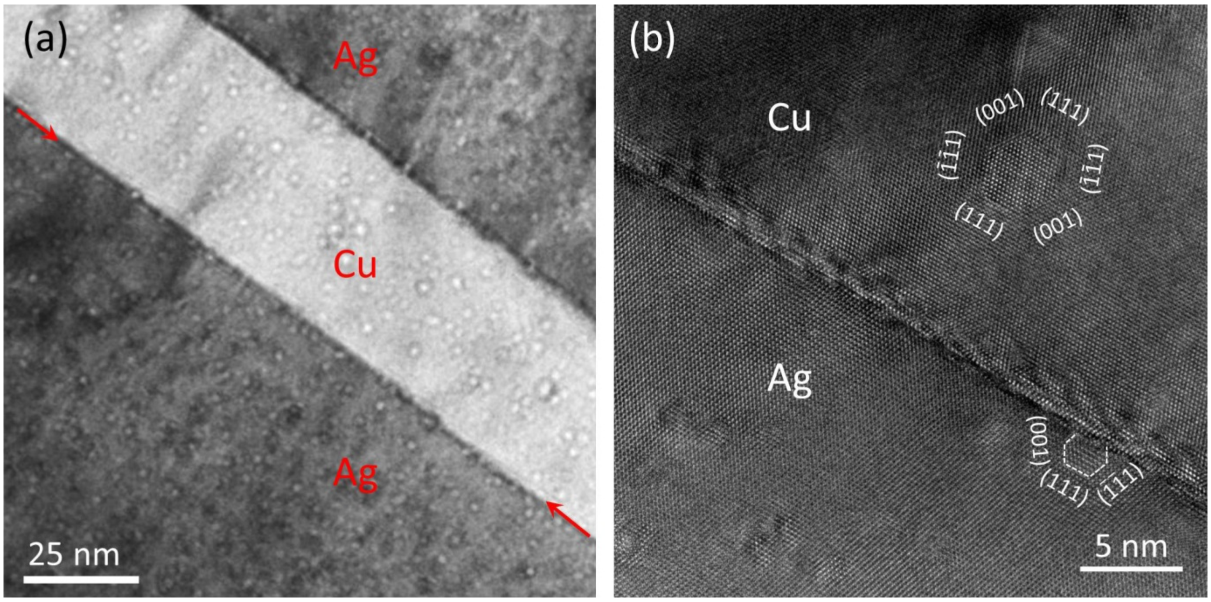

Supplementary Figure 3: (a) Under-focus (-1.5 µm) TEM image of the Cu-Ag composite after a He irradiation at 250 ºC; (b) polyhedron-shaped voids in both Cu and Ag.

To study the shapes of voids in Cu and Ag, irradiation was performed with the same He energy and dose as described in the text, but at a temperature of 250 ºC. Under these conditions, voids remain in the Cu and Ag layers. Figure 3a is an under-focus TEM image of the sample irradiated at 250 ºC, which shows voids in the interiors of Cu and Ag as well as at interfaces. Fig. 3b shows a high resolution TEM (HRTEM) image of the interface marked with arrows in supplementary Fig. 3a. In the Cu phase, a void lies away from the interface, while in the Ag phase, a void adheres to the interface. Voids in both the Cu and Ag phases have a truncated octahedron shape comprised of {111} and {100} planes. The octahedron shape follows the shape predicted by the Wulff construction to minimize surface free energy.
